# The Abnormal Sinus Rhythm: Myxedema Coma Complicated by Subacute Cardiac Tamponade

**DOI:** 10.7759/cureus.19535

**Published:** 2021-11-13

**Authors:** Lu Chen, Andrew V Doodnauth, Uta S Guo, Krunal H Patel, Yongxia S Qu, Cristina A Mitre

**Affiliations:** 1 Cardiology, State University of New York Downstate Medical Center, Brooklyn, USA; 2 Internal Medicine, State University of New York Downstate Medical Center, Brooklyn, USA; 3 Family Medicine and Community Health, Icahn School of Medicine at Mount Sinai, New York City, USA; 4 Cardiology, New York-Presbyterian Brooklyn Methodist Hospital, Brooklyn, USA; 5 Cardiology, Veterans Affairs New York Harbor Healthcare System, Brooklyn Campus, Brooklyn, USA

**Keywords:** hypothyroid, myxedema coma, hypothyroid pericardial effusion, pericardial effusion, cardiac tamponade

## Abstract

Subacute cardiac tamponade (SCT) is a potentially life-threatening condition that requires immediate medical attention. Combining careful history taking, focused physical exam, and the use of point of care ultrasound (POCUS) for early diagnosis with aggressive management can minimize potential complications. In patients with severe hypothyroidism and myxedema coma, clinical signs of cardiac tamponade may be masked and lead to delayed diagnosis. We present a case of a 67-year-old female with SCT secondary to myxedema coma, necessitating emergent pericardiocentesis following the identification of a large pericardial effusion with tamponade physiology. This case highlights the importance of thorough history taking with focused diagnostic workup, including POCUS in patients with an insidious presentation of SCT.

## Introduction

Myxedema coma is a rare complication of severe hypothyroidism that is potentially fatal [1]. Widespread use of thyroid hormone supplementation lead to diminished number of cases of myxedema coma. Today, the rarity of myxedema coma may subvert the physician’s differential diagnosis and delay the diagnosis of its potentially life-threatening complications. In a Taiwanese analysis, patients with primary overt hypothyroidism or myxedema coma are frequently missed in the emergency department [2]. Of note, 32% of the studied patients were found to have pericardial effusion, none with tamponade physiology. However, rare cases of cardiac tamponade has been reported with overt hypothyroidism [3-5]. 

Due to the slow rate of fluid accumulation, cardiac tamponade associated with myxedema coma or severe hypothyroidism is usually of subacute nature with insidious onset and subtle presentation. In addition to its rarity, the non-specific clinical signs, and the slow progression of pericardial effusion makes cardiac tamponade challenging to diagnose on initial hospitalization [3]. While the most common sign of cardiac tamponade is sinus tachycardia, tachycardia associated with myxedema coma is often masked, potentially delaying both diagnosis and treatment. We present the case of a patient with subtle subacute cardiac tamponade (SCT) secondary to myxedema coma to highlight its diagnostic challenges and potentials for improvement. 

## Case presentation

A 67-year-old female with a past medical history of iatrogenic hypothyroidism secondary to thyroidectomy stopped taking her levothyroxine over a year ago. Three months prior to her presentation, she had been experiencing progressively worsening confusion and respiratory distress. According to her family, she became bed-bound approximately a week before admission, when she was brought in by ambulance after being found unresponsive by family members. 

**Figure 1:**
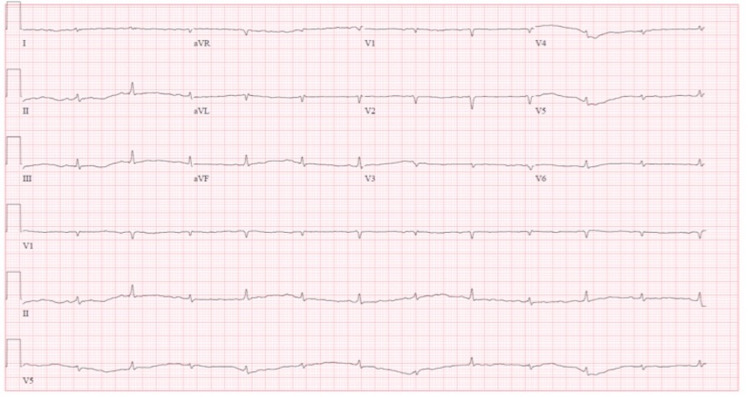
EKG on admission showing sinus rhythm with low voltage QRS complexes with electrical alternans EKG: electrocardiogram

**Figure 2:**
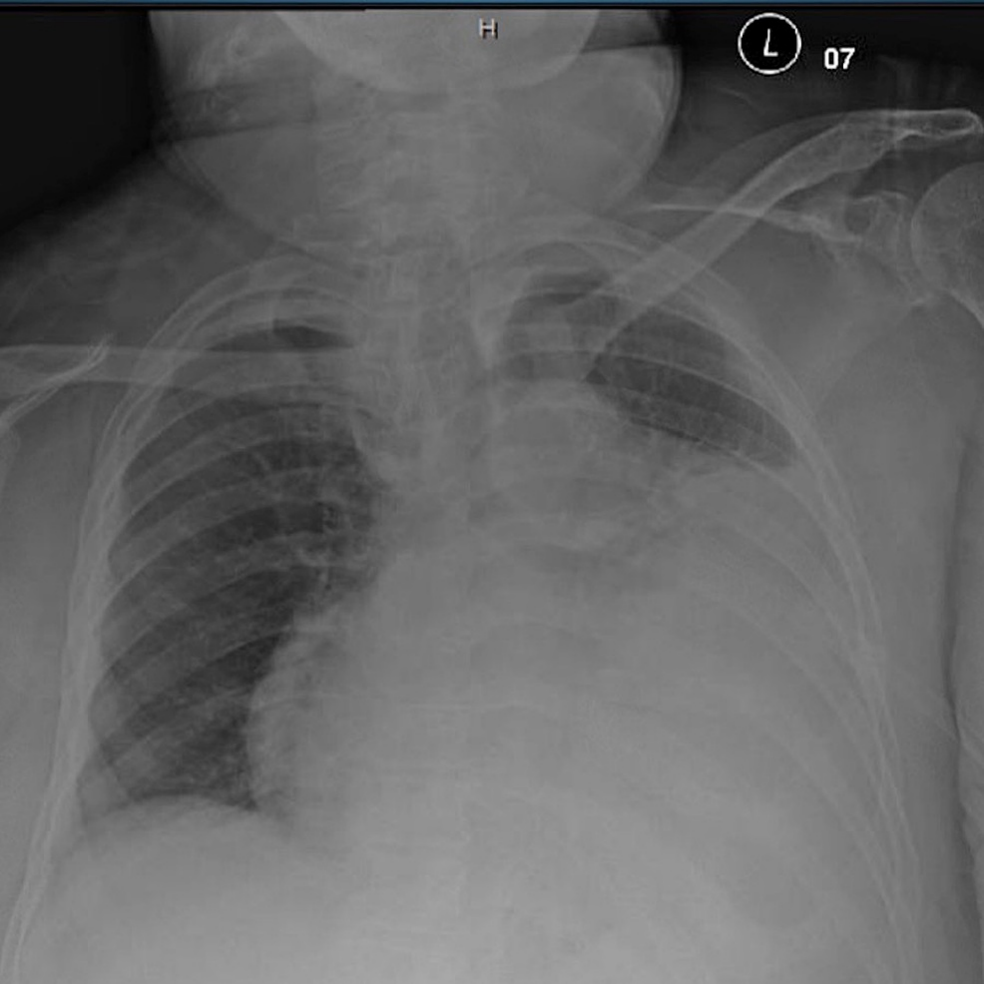
Admission chest radiography showing enlarged cardiac silhouette and congested lung fields

On admission, her vital signs were unremarkable with a heart rate of 70 beats per minute. Her physical examination was significant for lethargy, facial swelling, macroglossia, and bibasilar crackles of the lung fields. Laboratory findings were pertinent for thyroid-stimulating hormone (TSH) at 58.5 ng/dL and free thyroxine (T4) of 0.19 ng/dL. Electrocardiogram (EKG) showed normal sinus rhythm with low voltage QRS complexes with electrical alternans (Figure 1). The enlarged cardiac silhouette was noted on chest radiography (CXR) along with congested lung fields (Figure 2). Following consultation with the endocrinology service, the patient was started on intravenous levothyroxine and hydrocortisone and admitted to medicine telemetry service for further management.

**Figure 3:**
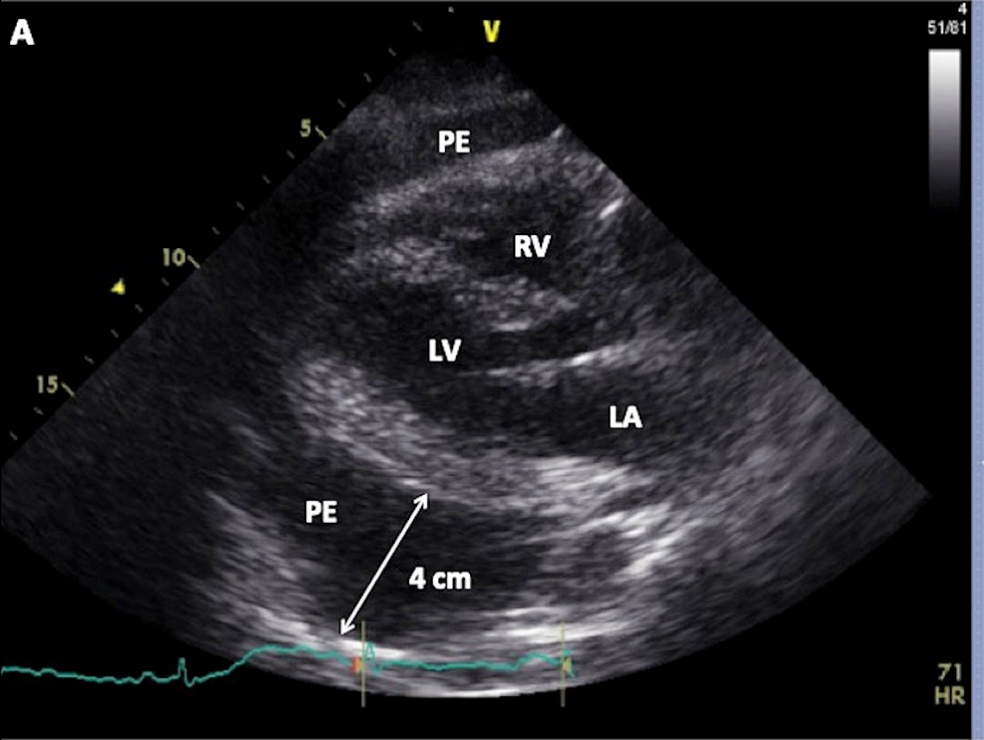
Echocardiographic image taken in the parasternal long axis view demonstrates a large pericardial effusion up to 4 cm PE: pericardial effusion; RV: right ventricle; LV: left ventricle; LA: left atrium.

**Figure 4:**
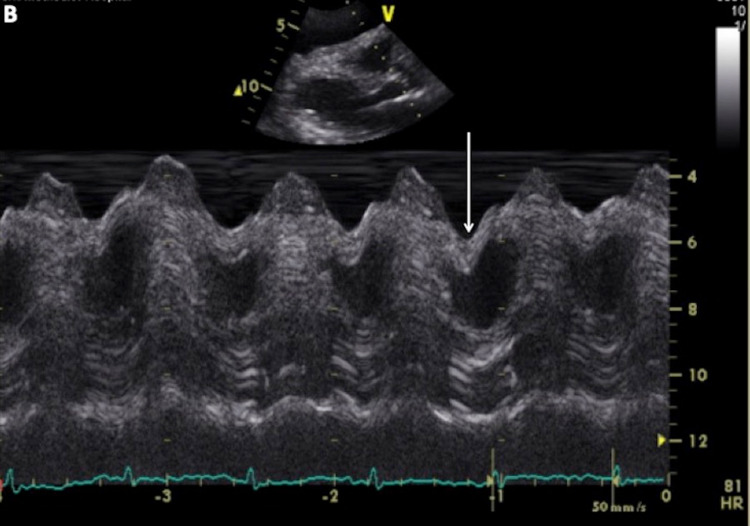
M-mode echocardiogram image in the parasternal long axis view demonstrates the right ventricle diastolic collapse (arrow)

**Figure 5:**
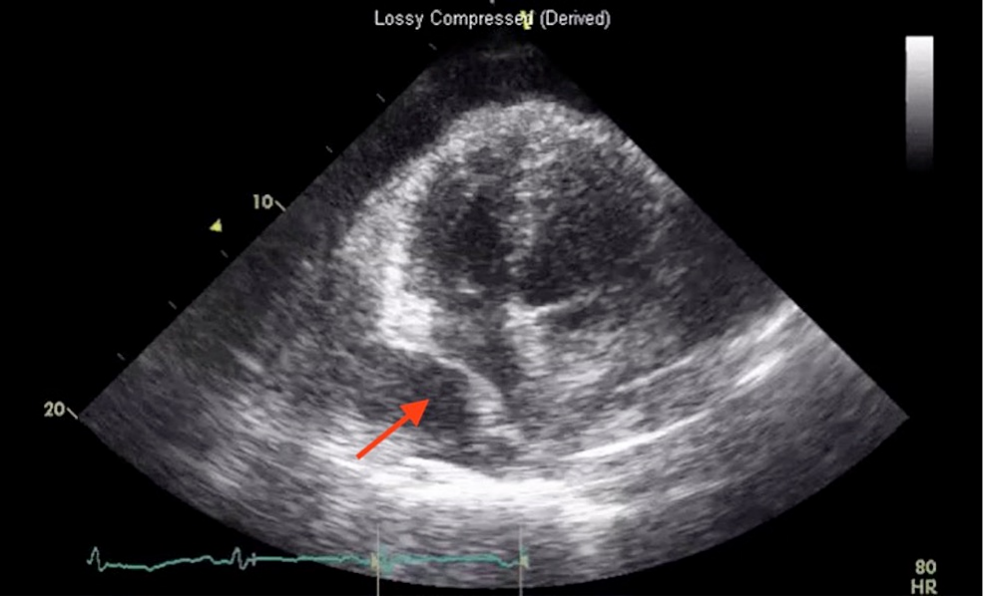
Apical four chambers view of ventricular systolic demonstrating right atrial collapse (arrow)

On the following day, a transthoracic echocardiogram (TTE) showed a large pericardial effusion with the swinging of the heart in the pericardial sac with the diastolic collapse of both atria and the right ventricle (Figure 3, Figure 4, Figure 5). The patient was upgraded to Cardiac ICU and underwent urgent pericardiocentesis with 1300 mL serous straw-colored fluid drained. Analysis of pericardial fluid was negative for malignant cells. Acid-fast bacilli stain and culture were negative. The patient was discharged on levothyroxine. Serial TSH and T4 measurements over the subsequent two months showed normalization of both values. On six months follow-up, repeat TTE showed diminishing pericardial effusion without echocardiographic evidence of cardiac tamponade.

## Discussion

The most challenging aspect of this case lies within recognizing elusive clues in a rare disease. On review of available literature, the only reported case of cardiac tamponade associated with myxedema coma was diagnosed after pericardial effusion was incidentally found on CT of the abdomen [4]. When cases of hypothyroidism associated cardiac tamponade were included, initial diagnoses made with CT scan were also noted [6-9]. Urgency in managing myxedema coma likely overshadowed diagnosis of associated complications such as SCT. In our patient with insidious onset of non-specific symptoms and subtle signs, myxedema coma was diagnosed with an initial screening laboratory study and managed appropriately. Nevertheless, SCT was overlooked and resulted in a delay in initial diagnosis.

In cases like myxedema coma, the lack of thyroid hormone can result in sinus bradycardia, decreased myocardial contractility, and fluid accumulation in serous cavities like the pericardial space [10]. Normal heart rate observed in our patient resulted from balanced bradycardia from myxedema coma and tachycardia from cardiac tamponade. The normal heart rate blunted expected clinical response from both conditions, resulting in the diagnostic challenge of this case. Despite its limited sensitivity, EKG findings have high specificity and can be used as an adjuvant test to rule in cardiac tamponade [11]. Sinus tachycardia is an appropriate compensatory mechanism of decreased cardiac output in the setting of tamponade physiology. While sinus tachycardia is commonly seen in patients presenting with cardiac tamponade, retrospective analysis showed only moderate sensitivity and positive predictive value. In our case, sinus tachycardia is likely blunted due to decreased sympathetic drive from lack of thyroid hormone [12].

Clues overlooked in the EKG of our patient include the minimal voltage of QRS complexes and electrical alternans. Low QRS voltage (LQRSV) can be a result of low voltage potential generated at cardiac myocytes, such as infiltrative cardiomyopathy and extensive myocardial infarction. Extra-cardiac causes of LQRSV can be from large pericardial effusion, extreme obesity, and chronic obstructive lung disease due to increase interference of electrical conduction [13]. When LQRSV is seen in combination of electrical alternans, suspicion increases for cardiac tamponade with large pericardial effusion [11].

In addition to an abnormal EKG, an enlarged cardiac silhouette on CXR can also provide a clue to prompt further diagnostic workup of SCT. Radiological studies are often utilized in the emergency department for an expedited and generalized evaluation of cardiopulmonary disease. CXR has low sensitivity in patients with post-operative cardiac tamponade when compared to pre-operative CXR [14]. A relative increase in the size of the cardiac silhouette, especially acutely, leads to increased suspicion of pericardial effusion. While CXR can provide anatomical information, it lacks hemodynamic assessment, thus cannot be used to differentiate pericardial effusion from cardiac tamponade.

Ultimately, the diagnosis of SCT in this case was made with echocardiography on the following day. TTE is the gold standard to confirm the diagnosis of cardiac tamponade. While standard TTE provides detailed and accurate diagnosis for a patient with tamponade, availability, especially outside of regular business hours, can be an issue. In the emergency department, where time is of premium, bedside point of care ultrasound (POCUS) can help rule in the diagnosis to facilitate appropriate triage decisions and shorten time to definitive therapy as well as length of stay [15]. In our case, POCUS can be used to rapidly exclude pericardial as well as pleural effusion. While differentiating between pericardial effusion and cardiac tamponade may be complex, large pericardial effusion will likely trigger timely cardiology consultation and facilitate appropriate triaging decisions. In our patient with myxedema coma, the abnormal EKG with low voltage and electrical alternans should have prompted an earlier echocardiographic evaluation with TTE or at least POCUS, with appropriate cardiac consult to avoid an outcome that could have been potentially fatal.

The treatment of pericardial effusion will vary depending upon the underlying etiology and, most notably, the presence or absence of cardiac tamponade. In cases of tamponade physiology, emergent drainage of pericardial effusion is indicated. In patients with SCT from myxedema coma, thyroid hormone replacement therapy with appropriate clinical support should be provided simultaneously. A timely diagnosis of both myxedema coma and tamponade physiology is critical for successful treatment in this case.

## Conclusions

Both cardiac tamponade and myxedema coma are life-threatening conditions alone. Our case highlights the importance of careful history taking supplemented by appropriate diagnostic testing while evaluating a patient with insidious SCT. Patients with severe hypothyroidism may not present with classic signs and symptoms of cardiac tamponade, such as tachycardia. Therefore a high index of suspicion is required to diagnose this life-threatening complication of hypothyroidism. Perhaps expedited POCUS or TTE should be considered routinely upon initial evaluation in patients with severe hypothyroidism or myxedema coma, so prompt diagnosis can lead to complete recovery. 

## References

[1] Wall CR (2000). Myxedema coma: diagnosis and treatment. Am Fam Physician.

[2] Chen YJ, Hou SK, How CK (2010). Diagnosis of unrecognized primary overt hypothyroidism in the ED. Am J Emerg Med.

[3] Ekka M, Ali I, Aggarwal P, Jamshed N (2014). Cardiac tamponade as initial presenting feature of primary hypothyroidism in the ED. Am J Emerg Med.

[4] Majid-Moosa A, Schussler JM, Mora A (2015). Myxedema coma with cardiac tamponade and severe cardiomyopathy. Proc (Bayl Univ Med Cent).

[5] MA L, SP GS (1965). Case of myxoedema with a huge pericardial effusion and cardiac tamponade. Br Med J.

[6] Almani MU, Usman M, Arif AW, Ayub MT, Fatima N (2020). Rare presentation of cardiac tamponade in a patient with subclinical hypothyroidism. Cureus.

[7] Garcia S, Tin SL (2020). SAT-484 presyncope as initial presentation of massive pericardial effusion with tamponade in a patient with primary hypothyroidism. J Endocr Soc.

[8] Schmitt W, Roque D, Germano A (2018). Massive pericardial effusion caused by hypothyroidism. Clin Case Rep.

[9] Motabar A, Anousheh R, Shaker R, Pai RG (2011). A rare case of amiodarone-induced hypothyroidism presenting with cardiac tamponade. Int J Angiol.

[10] Klein I, Danzi S (2016). Thyroid disease and the heart. Curr Probl Cardiol.

[11] Ang KP, Nordin RB, Lee SCY, Lee CY, Lu HT (2019). Diagnostic value of electrocardiogram in cardiac tamponade. Med J Malaysia.

[12] Nicoloff JT, LoPresti JS (1993). Myxedema coma. A form of decompensated hypothyroidism. Endocrinol Metab Clin North Am.

[13] Madias JE (2008). Low QRS voltage and its causes. J Electrocardiol.

[14] Hamid M, Khan MU, Bashour AC (2006). Diagnostic value of chest X-ray and echocardiography for cardiac tamponade in post cardiac surgery patients. JPMA J Pak Med Assoc.

[15] Alpert EA, Amit U, Guranda L, Mahagna R, Grossman SA, Bentancur A (2017). Emergency department point-of-care ultrasonography improves time to pericardiocentesis for clinically significant effusions. Clin Exp Emerg Med.

